# 4-Acetyl-3-(*p*-anis­yl)sydnone

**DOI:** 10.1107/S1600536811018484

**Published:** 2011-05-25

**Authors:** Hoong-Kun Fun, Wan-Sin Loh, Nithinchandra, Balakrishna Kalluraya

**Affiliations:** a X-ray Crystallography Unit, School of Physics, Universiti Sains Malaysia, 11800 USM, Penang, Malaysia; b Department of Studies in Chemistry, Mangalore University, Mangalagangotri, Mangalore 574 199, India

## Abstract

The asymmetric unit of the title compound [systematic name: 4-acetyl-3-(4-meth­oxy­phen­yl)-1,2,3-oxadiazol-3-ium-5-olate], C_11_H_10_N_2_O_4_, contains four crystallographically independent mol­ecules. The 1,2,3-oxadiazole rings are almost planar [maximum deviations = 0.006 (3), 0.006 (3), 0.002 (3) and 0.009 (3) Å] and form dihedral angles of 55.03 (14), 61.02 (13), 58.36 (14) and 53.79 (15)° with their attached benzene rings. In the crystal, inter­molecular C—H⋯O and C—H⋯N hydrogen bonds link the mol­ecules, forming sheets parallel to (011).

## Related literature

For background to sydnones, see: Rai *et al.* (2008 ▶); Hedge *et al.* (2008 ▶). For a related structure, see: Fun *et al.* (2010 ▶). For bond-length data, see: Allen *et al.* (1987 ▶). For the stability of the temperature controller used in the data collection, see: Cosier & Glazer (1986 ▶).
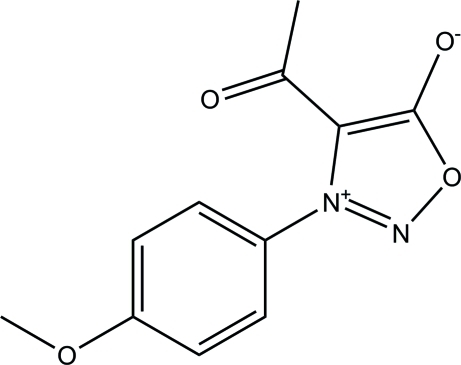


## Experimental

### 

#### Crystal data

C_11_H_10_N_2_O_4_
*M* *_r_* = 234.21
Triclinic, 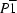

*a* = 9.6338 (8) Å
*b* = 10.0679 (7) Å
*c* = 22.4108 (18) Å
α = 86.938 (3)°
β = 78.049 (2)°
γ = 89.891 (2)°
*V* = 2123.4 (3) Å^3^
*Z* = 8
Mo *K*α radiation
μ = 0.11 mm^−1^
*T* = 100 K
0.53 × 0.17 × 0.08 mm

#### Data collection

Bruker SMART APEXII DUO CCD area-detector diffractometer
Absorption correction: multi-scan (*SADABS*; Bruker, 2009 ▶) *T*_min_ = 0.942, *T*_max_ = 0.991
36725 measured reflections
9556 independent reflections
7589 reflections with *I* > 2σ(*I*)
*R*_int_ = 0.044

#### Refinement

*R*[*F* ^2^ > 2σ(*F* ^2^)] = 0.052
*wR*(*F* ^2^) = 0.155
*S* = 1.04
9556 reflections
622 parameters
H-atom parameters constrained
Δρ_max_ = 0.32 e Å^−3^
Δρ_min_ = −0.29 e Å^−3^

### 

Data collection: *APEX2* (Bruker, 2009 ▶); cell refinement: *SAINT* (Bruker, 2009 ▶); data reduction: *SAINT*; program(s) used to solve structure: *SHELXTL* (Sheldrick, 2008 ▶); program(s) used to refine structure: *SHELXTL*; molecular graphics: *SHELXTL*; software used to prepare material for publication: *SHELXTL* and *PLATON* (Spek, 2009 ▶).

## Supplementary Material

Crystal structure: contains datablocks global, I. DOI: 10.1107/S1600536811018484/rz2595sup1.cif

Structure factors: contains datablocks I. DOI: 10.1107/S1600536811018484/rz2595Isup2.hkl

Supplementary material file. DOI: 10.1107/S1600536811018484/rz2595Isup3.cml

Additional supplementary materials:  crystallographic information; 3D view; checkCIF report

## Figures and Tables

**Table 1:** Hydrogen-bond geometry (Å, °)

*D*—H⋯*A*	*D*—H	H⋯*A*	*D*⋯*A*	*D*—H⋯*A*
C5*A*—H5*AA*⋯O3*B*^i^	0.93	2.49	3.282 (3)	144
C4*B*—H4*BA*⋯O3*A*	0.93	2.48	3.217 (3)	137
C8*B*—H8*BA*⋯N2*A*^ii^	0.93	2.47	3.369 (4)	164
C4*C*—H4*CA*⋯O3*D*^iii^	0.93	2.49	3.231 (3)	137
C11*B*—H11*D*⋯O2*D*^ii^	0.96	2.39	3.220 (4)	144
C11*C*—H11*G*⋯O2*B*^iv^	0.96	2.47	3.289 (4)	143
C8*C*—H8*CA*⋯N2*D*^v^	0.93	2.47	3.386 (4)	169
C5*D*—H5*DA*⋯O3*C*^vi^	0.93	2.56	3.311 (3)	138

Symmetry codes: (i) 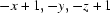; (ii) 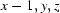; (iii) 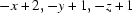; (iv) 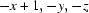; (v) 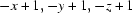; (vi) 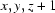.

**Fig. 1.**
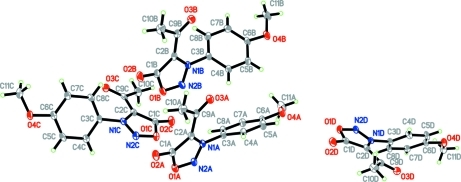
The molecular structure of the title compound, showing 50% probability displacement ellipsoids.

**Fig. 2.**
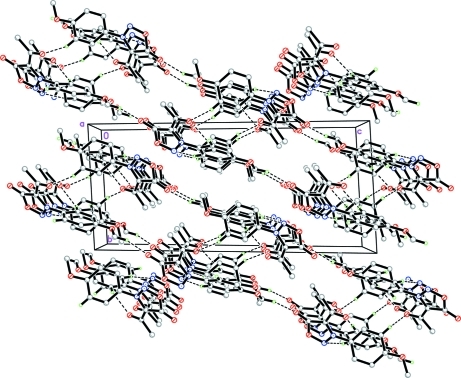
The crystal packing of the title compound, viewed along the a axis. H atoms not involved in the intermolecular interactions (dashed lines) are omitted for clarity.

## supplementary crystallographic information

### Comment

Sydnones constitute a well defined class of mesoionic compounds that contain the 1,2,3-oxadiazole ring system. The introduction of the concept of mesoionic structure for certain heterocyclic compounds in the year 1949 has proved to be a fruitful development in heterocyclic chemistry. The study of sydnones still remains a field of interest because of their electronic structure and also because of the various types of biological activities displayed by some of them. Interest in sydnone derivatives has also been encouraged by the discovery that they exhibit various pharmacological activities (Hedge *et al.*, 2008; Rai *et al.*, 2008). The title 4-acetyl sydnone will be used for the preparation of a new series of α,β-unsaturated carbonyl compounds (namely chalcones) by condensation with appropriate ketones or aldehydes. These α,β-unsaturated carbonyl compounds will be utilized for the synthesis of a variety of novel heterocyclic compounds like pyrazolines, pyrazole *etc*, carrying sydnone moiety.

The asymmetric unit of the title compound (Fig. 1) consists of four crystallographically independent molecules (molecule *A*, *B*, *C* & *D*). The 1,2,3-oxadiazole (O1/N1/N2/C1/C2) rings are almost planar [maximum deviations of 0.006 (3) Å at atom N2A; 0.006 (3) Å at atom C1B; 0.002 (3) Å at atom C1C and 0.009 (3) Å at N2D] and form dihedral angles of 55.03 (14), 61.02 (13), 58.36 (14) and 53.79 (15)° with their attached benzene rings (C3–C8). The bond lengths (Allen *et al.*, 1987) and angles are within the normal ranges and are comparable to those found in a related structure (Fun *et al.*, 2010).

In the crystal packing (Fig. 2), intermolecular C5A—H5AA···O3B, C4B—H4BA···O3A, C8B—H8BA···N2A, C4C—H4CA···O3D, C11B—H11D···O2D, C11C—H11G···O2B, C8C—H8CA···N2D and C5D—H5DA···O3C hydrogen bonds (Table 1) link the molecules to form two-dimensional sheets parallel to the (011) plane.

### Experimental

To a suspension of phosphorous pentoxide (0.1 mol) in benzene (100 ml) contained in a three-necked round-bottom flask fitted with a reflux condenser, 3-(*p*-anisyl)sydnone (0.05 mol) was added. The mixture was heated to reflux on a water bath. Glacial acetic acid (0.05 mol) was added dropwise through the dropping funnel over a ten minutes period. The reaction mixture heated for 4 h. After cooling to room temperature, the benzene was decanted and evaporated to dryness. The solid obtained was filtered, dried and recrystallized from ethanol. Single crystals suitable for X-ray analysis were obtained from a mixture of DMF and ethanol (1:2 *v*/*v*) by slow evaporation.

### Refinement

All H atoms were positioned geometrically and refined using a riding model with *U*_iso_(H) = 1.2 or 1.5 *U*_eq_(C) [C–H = 0.93 to 0.96 Å]. A rotating group model was applied to the methyl groups. The crystal is a twin with twin law 1 0 0, 0 - 1 0, 1 0 -1 and BASF = 0.198 (2). Nine outliners were omitted for the final refinement, 2 0 4, 5 1 4, 5 2 1, 5 -2 8, 3 0 5, 2 3 5, -6 -2 5, 5 -4 6 and 5 -3 12.

### Crystal data

**Table:**

C_11_H_10_N_2_O_4_	*Z* = 8
*M**_r_* = 234.21	*F*(000) = 976
Triclinic, *P*1	*D*_x_ = 1.465 Mg m^−^^3^
Hall symbol: -P 1	Mo *K*α radiation, λ = 0.71073 Å
*a* = 9.6338 (8) Å	Cell parameters from 8454 reflections
*b* = 10.0679 (7) Å	θ = 3.2–32.4°
*c* = 22.4108 (18) Å	µ = 0.11 mm^−^^1^
α = 86.938 (3)°	*T* = 100 K
β = 78.049 (2)°	Block, colourless
γ = 89.891 (2)°	0.53 × 0.17 × 0.08 mm
*V* = 2123.4 (3) Å^3^	

### Data collection

**Table:**

Bruker SMART APEXII DUO CCD area-detector diffractometer	9556 independent reflections
Radiation source: fine-focus sealed tube	7589 reflections with *I* > 2σ(*I*)
graphite	*R*_int_ = 0.044
φ and ω scans	θ_max_ = 27.5°, θ_min_ = 0.9°
Absorption correction: multi-scan (*SADABS*; Bruker, 2009)	*h* = −12→12
*T*_min_ = 0.942, *T*_max_ = 0.991	*k* = −13→13
36725 measured reflections	*l* = −29→29

### Refinement

**Table:**

Refinement on *F*^2^	Primary atom site location: structure-invariant direct methods
Least-squares matrix: full	Secondary atom site location: difference Fourier map
*R*[*F*^2^ > 2σ(*F*^2^)] = 0.052	Hydrogen site location: inferred from neighbouring sites
*wR*(*F*^2^) = 0.155	H-atom parameters constrained
*S* = 1.04	*w* = 1/[σ^2^(*F*_o_^2^) + (0.0694*P*)^2^ + 1.9792*P*] where *P* = (*F*_o_^2^ + 2*F*_c_^2^)/3
9556 reflections	(Δ/σ)_max_ = 0.001
622 parameters	Δρ_max_ = 0.32 e Å^−^^3^
0 restraints	Δρ_min_ = −0.29 e Å^−^^3^

### Special details

**Table:**

Experimental. The crystal was placed in the cold stream of an Oxford Cryosystems Cobra open-flow nitrogen cryostat (Cosier & Glazer, 1986) operating at 100.0 (1) K.
Geometry. All e.s.d.'s (except the e.s.d. in the dihedral angle between two l.s. planes) are estimated using the full covariance matrix. The cell e.s.d.'s are taken into account individually in the estimation of e.s.d.'s in distances, angles and torsion angles; correlations between e.s.d.'s in cell parameters are only used when they are defined by crystal symmetry. An approximate (isotropic) treatment of cell e.s.d.'s is used for estimating e.s.d.'s involving l.s. planes.
Refinement. Refinement of *F*^2^ against ALL reflections. The weighted *R*-factor *wR* and goodness of fit *S* are based on *F*^2^, conventional *R*-factors *R* are based on *F*, with *F* set to zero for negative *F*^2^. The threshold expression of *F*^2^ > σ(*F*^2^) is used only for calculating *R*-factors(gt) *etc*. and is not relevant to the choice of reflections for refinement. *R*-factors based on *F*^2^ are statistically about twice as large as those based on *F*, and *R*- factors based on ALL data will be even larger.

### Fractional atomic coordinates and isotropic or equivalent isotropic displacement parameters (Å^2^)

**Table:**

	*x*	*y*	*z*	*U*_iso_*/*U*_eq_	
O1A	1.0268 (2)	0.15731 (17)	0.25992 (8)	0.0198 (4)	
O2A	0.9645 (2)	−0.02461 (18)	0.21501 (8)	0.0235 (4)	
O3A	0.6205 (2)	−0.01215 (17)	0.37893 (8)	0.0208 (4)	
O4A	0.6496 (2)	0.30248 (18)	0.57834 (8)	0.0246 (4)	
N1A	0.8745 (2)	0.15265 (19)	0.34312 (9)	0.0150 (4)	
N2A	0.9836 (2)	0.2203 (2)	0.31319 (9)	0.0180 (4)	
C1A	0.9400 (3)	0.0439 (2)	0.25844 (11)	0.0183 (5)	
C2A	0.8392 (3)	0.0448 (2)	0.31472 (11)	0.0162 (5)	
C3A	0.8143 (3)	0.1951 (2)	0.40338 (10)	0.0150 (5)	
C8A	0.7703 (3)	0.3254 (2)	0.41043 (11)	0.0177 (5)	
H8AA	0.7765	0.3847	0.3767	0.021*	
C7A	0.7164 (3)	0.3658 (2)	0.46930 (11)	0.0192 (5)	
H7AA	0.6882	0.4533	0.4752	0.023*	
C6A	0.7047 (3)	0.2748 (2)	0.51937 (11)	0.0191 (5)	
C5A	0.7513 (3)	0.1440 (2)	0.51073 (11)	0.0225 (6)	
H5AA	0.7448	0.0839	0.5442	0.027*	
C4A	0.8069 (3)	0.1040 (2)	0.45262 (11)	0.0200 (5)	
H4AA	0.8387	0.0175	0.4467	0.024*	
C9A	0.7135 (3)	−0.0400 (2)	0.33564 (11)	0.0173 (5)	
C10A	0.7041 (3)	−0.1618 (3)	0.30084 (12)	0.0247 (6)	
H10A	0.6142	−0.2053	0.3161	0.037*	
H10B	0.7792	−0.2215	0.3059	0.037*	
H10C	0.7129	−0.1366	0.2583	0.037*	
C11A	0.5980 (4)	0.4332 (3)	0.59009 (13)	0.0317 (7)	
H11A	0.5618	0.4392	0.6331	0.048*	
H11B	0.5235	0.4521	0.5684	0.048*	
H11C	0.6739	0.4965	0.5767	0.048*	
O1B	0.5532 (2)	0.17633 (18)	0.26742 (8)	0.0222 (4)	
O2B	0.4936 (2)	0.0067 (2)	0.21410 (8)	0.0271 (4)	
O3B	0.1346 (2)	−0.00518 (17)	0.37277 (8)	0.0203 (4)	
O4B	0.1537 (2)	0.31158 (19)	0.58119 (8)	0.0243 (4)	
N1B	0.3873 (2)	0.16804 (19)	0.34621 (9)	0.0153 (4)	
N2B	0.5034 (2)	0.2329 (2)	0.32200 (9)	0.0205 (5)	
C1B	0.4621 (3)	0.0700 (3)	0.25912 (11)	0.0193 (5)	
C2B	0.3522 (3)	0.0686 (2)	0.31215 (10)	0.0164 (5)	
C3B	0.3205 (3)	0.2046 (2)	0.40707 (10)	0.0159 (5)	
C4B	0.4001 (3)	0.1923 (2)	0.45223 (11)	0.0168 (5)	
H4BA	0.4920	0.1594	0.4435	0.020*	
C5B	0.3403 (3)	0.2297 (2)	0.51025 (11)	0.0192 (5)	
H5BA	0.3920	0.2225	0.5410	0.023*	
C6B	0.2025 (3)	0.2783 (2)	0.52254 (11)	0.0178 (5)	
C7B	0.1239 (3)	0.2905 (2)	0.47659 (11)	0.0203 (5)	
H7BA	0.0318	0.3229	0.4852	0.024*	
C8B	0.1845 (3)	0.2540 (2)	0.41794 (11)	0.0185 (5)	
H8BA	0.1342	0.2626	0.3867	0.022*	
C9B	0.2298 (3)	−0.0198 (2)	0.32851 (11)	0.0173 (5)	
C10B	0.2272 (3)	−0.1319 (3)	0.28691 (13)	0.0286 (6)	
H10D	0.1389	−0.1798	0.2992	0.043*	
H10E	0.3043	−0.1910	0.2894	0.043*	
H10F	0.2366	−0.0964	0.2456	0.043*	
C11B	0.0120 (3)	0.3620 (3)	0.59641 (13)	0.0314 (7)	
H11D	−0.0097	0.3826	0.6386	0.047*	
H11E	−0.0538	0.2961	0.5894	0.047*	
H11F	0.0047	0.4410	0.5714	0.047*	
O1C	0.8181 (2)	0.34109 (18)	0.22984 (8)	0.0215 (4)	
O2C	0.6999 (2)	0.50964 (18)	0.28147 (8)	0.0248 (4)	
O3C	0.4962 (2)	0.50729 (17)	0.12143 (8)	0.0201 (4)	
O4C	0.7211 (2)	0.17885 (18)	−0.08198 (8)	0.0218 (4)	
N1C	0.7281 (2)	0.34063 (19)	0.15082 (9)	0.0150 (4)	
N2C	0.8225 (2)	0.2802 (2)	0.17627 (9)	0.0193 (4)	
C1C	0.7148 (3)	0.4441 (2)	0.23698 (11)	0.0185 (5)	
C2C	0.6572 (3)	0.4402 (2)	0.18356 (11)	0.0165 (5)	
C3C	0.7212 (3)	0.2989 (2)	0.09044 (10)	0.0149 (5)	
C4C	0.8447 (3)	0.3067 (2)	0.04600 (11)	0.0174 (5)	
H4CA	0.9284	0.3392	0.0545	0.021*	
C5C	0.8406 (3)	0.2648 (2)	−0.01181 (11)	0.0192 (5)	
H5CA	0.9223	0.2688	−0.0424	0.023*	
C6C	0.7146 (3)	0.2170 (2)	−0.02389 (11)	0.0172 (5)	
C7C	0.5916 (3)	0.2098 (2)	0.02156 (11)	0.0189 (5)	
H7CA	0.5074	0.1781	0.0132	0.023*	
C8C	0.5956 (3)	0.2503 (2)	0.07946 (11)	0.0187 (5)	
H8CA	0.5146	0.2448	0.1104	0.022*	
C9C	0.5472 (3)	0.5250 (2)	0.16617 (11)	0.0167 (5)	
C10C	0.4988 (3)	0.6369 (3)	0.20708 (12)	0.0254 (6)	
H10G	0.4172	0.6780	0.1958	0.038*	
H10H	0.5738	0.7015	0.2028	0.038*	
H10I	0.4743	0.6025	0.2487	0.038*	
C11C	0.5948 (3)	0.1238 (3)	−0.09556 (12)	0.0279 (6)	
H11G	0.6142	0.0969	−0.1369	0.042*	
H11H	0.5216	0.1896	−0.0906	0.042*	
H11I	0.5640	0.0480	−0.0682	0.042*	
O1D	0.7154 (2)	0.66298 (18)	0.76043 (8)	0.0205 (4)	
O2D	0.8187 (2)	0.47850 (19)	0.71668 (8)	0.0266 (4)	
O3D	1.0010 (2)	0.48713 (17)	0.88251 (8)	0.0202 (4)	
O4D	0.7767 (2)	0.80695 (17)	1.08041 (8)	0.0215 (4)	
N1D	0.7863 (2)	0.65663 (19)	0.84444 (9)	0.0150 (4)	
N2D	0.7072 (2)	0.7257 (2)	0.81382 (9)	0.0187 (4)	
C1D	0.8016 (3)	0.5472 (2)	0.75995 (11)	0.0193 (5)	
C2D	0.8471 (3)	0.5475 (2)	0.81658 (11)	0.0172 (5)	
C3D	0.7877 (3)	0.6988 (2)	0.90516 (10)	0.0152 (5)	
C4D	0.7475 (3)	0.6070 (2)	0.95461 (11)	0.0202 (5)	
H4DA	0.7222	0.5204	0.9486	0.024*	
C5D	0.7463 (3)	0.6473 (2)	1.01240 (11)	0.0213 (5)	
H5DA	0.7212	0.5871	1.0458	0.026*	
C6D	0.7827 (3)	0.7790 (2)	1.02127 (11)	0.0166 (5)	
C7D	0.8199 (3)	0.8698 (2)	0.97135 (11)	0.0177 (5)	
H7DA	0.8423	0.9573	0.9772	0.021*	
C8D	0.8236 (3)	0.8285 (2)	0.91230 (11)	0.0176 (5)	
H8DA	0.8497	0.8877	0.8785	0.021*	
C9D	0.9507 (3)	0.4609 (2)	0.83869 (11)	0.0174 (5)	
C10D	0.9925 (3)	0.3386 (3)	0.80422 (13)	0.0271 (6)	
H10J	1.0641	0.2920	0.8209	0.041*	
H10M	1.0292	0.3636	0.7620	0.041*	
H10K	0.9109	0.2818	0.8077	0.041*	
C11D	0.8095 (3)	0.9402 (3)	1.09304 (12)	0.0275 (6)	
H11M	0.8006	0.9464	1.1363	0.041*	
H11J	0.7449	1.0008	1.0788	0.041*	
H11K	0.9049	0.9626	1.0725	0.041*	

### Atomic displacement parameters (Å^2^)

**Table:**

	*U*^11^	*U*^22^	*U*^33^	*U*^12^	*U*^13^	*U*^23^
O1A	0.0245 (10)	0.0194 (9)	0.0140 (8)	0.0008 (7)	−0.0003 (7)	−0.0030 (7)
O2A	0.0316 (11)	0.0234 (9)	0.0149 (8)	0.0050 (8)	−0.0019 (8)	−0.0060 (7)
O3A	0.0244 (10)	0.0190 (9)	0.0181 (9)	−0.0013 (7)	−0.0022 (8)	−0.0020 (7)
O4A	0.0376 (12)	0.0220 (9)	0.0122 (8)	−0.0036 (8)	0.0005 (8)	−0.0043 (7)
N1A	0.0193 (11)	0.0133 (9)	0.0122 (9)	0.0000 (8)	−0.0027 (8)	−0.0003 (7)
N2A	0.0219 (11)	0.0196 (10)	0.0120 (9)	0.0000 (8)	−0.0024 (8)	−0.0025 (8)
C1A	0.0221 (13)	0.0172 (11)	0.0158 (12)	0.0025 (10)	−0.0044 (10)	−0.0006 (9)
C2A	0.0230 (13)	0.0122 (10)	0.0136 (11)	0.0029 (9)	−0.0037 (10)	−0.0033 (8)
C3A	0.0183 (12)	0.0136 (11)	0.0131 (11)	−0.0023 (9)	−0.0030 (9)	−0.0024 (8)
C8A	0.0235 (13)	0.0144 (11)	0.0150 (11)	−0.0015 (9)	−0.0038 (10)	0.0003 (9)
C7A	0.0250 (14)	0.0143 (11)	0.0181 (12)	−0.0022 (9)	−0.0041 (10)	−0.0019 (9)
C6A	0.0249 (14)	0.0200 (12)	0.0120 (11)	−0.0059 (10)	−0.0028 (10)	−0.0023 (9)
C5A	0.0354 (16)	0.0170 (12)	0.0152 (12)	−0.0043 (11)	−0.0056 (11)	0.0016 (9)
C4A	0.0303 (15)	0.0144 (11)	0.0158 (11)	−0.0011 (10)	−0.0059 (10)	−0.0017 (9)
C9A	0.0236 (13)	0.0149 (11)	0.0146 (11)	0.0022 (10)	−0.0067 (10)	−0.0001 (9)
C10A	0.0328 (16)	0.0188 (12)	0.0240 (13)	−0.0026 (11)	−0.0076 (12)	−0.0076 (10)
C11A	0.0448 (19)	0.0238 (14)	0.0227 (13)	−0.0002 (13)	0.0039 (13)	−0.0090 (11)
O1B	0.0245 (10)	0.0272 (9)	0.0129 (8)	−0.0004 (8)	0.0016 (7)	−0.0025 (7)
O2B	0.0320 (11)	0.0350 (11)	0.0139 (9)	0.0091 (9)	−0.0017 (8)	−0.0089 (8)
O3B	0.0222 (10)	0.0190 (8)	0.0195 (9)	−0.0008 (7)	−0.0035 (8)	−0.0019 (7)
O4B	0.0283 (11)	0.0299 (10)	0.0127 (8)	−0.0008 (8)	0.0020 (8)	−0.0064 (7)
N1B	0.0203 (11)	0.0146 (9)	0.0106 (9)	−0.0015 (8)	−0.0023 (8)	−0.0011 (7)
N2B	0.0209 (12)	0.0243 (11)	0.0139 (10)	−0.0032 (9)	0.0016 (9)	−0.0008 (8)
C1B	0.0233 (14)	0.0218 (12)	0.0132 (11)	0.0046 (10)	−0.0044 (10)	−0.0016 (9)
C2B	0.0234 (13)	0.0154 (11)	0.0112 (10)	0.0028 (9)	−0.0043 (10)	−0.0034 (8)
C3B	0.0229 (13)	0.0128 (10)	0.0112 (11)	−0.0026 (9)	−0.0013 (10)	−0.0022 (8)
C4B	0.0202 (13)	0.0146 (11)	0.0152 (11)	−0.0011 (9)	−0.0028 (9)	−0.0012 (9)
C5B	0.0279 (14)	0.0176 (12)	0.0126 (11)	−0.0036 (10)	−0.0057 (10)	0.0001 (9)
C6B	0.0246 (14)	0.0152 (11)	0.0120 (11)	−0.0045 (10)	0.0009 (10)	−0.0048 (9)
C7B	0.0210 (13)	0.0184 (12)	0.0208 (12)	0.0006 (10)	−0.0021 (10)	−0.0039 (9)
C8B	0.0246 (14)	0.0158 (11)	0.0164 (11)	−0.0015 (10)	−0.0064 (10)	−0.0025 (9)
C9B	0.0230 (13)	0.0145 (11)	0.0157 (11)	0.0033 (9)	−0.0068 (10)	−0.0015 (9)
C10B	0.0368 (17)	0.0208 (13)	0.0290 (15)	−0.0020 (12)	−0.0065 (13)	−0.0106 (11)
C11B	0.0322 (16)	0.0357 (16)	0.0217 (13)	0.0002 (13)	0.0070 (12)	−0.0093 (11)
O1C	0.0273 (10)	0.0246 (9)	0.0131 (8)	0.0010 (8)	−0.0052 (7)	−0.0017 (7)
O2C	0.0361 (12)	0.0242 (9)	0.0142 (8)	−0.0034 (8)	−0.0041 (8)	−0.0043 (7)
O3C	0.0232 (10)	0.0193 (9)	0.0181 (9)	0.0028 (7)	−0.0043 (8)	−0.0023 (7)
O4C	0.0273 (10)	0.0258 (9)	0.0121 (8)	0.0026 (8)	−0.0032 (7)	−0.0042 (7)
N1C	0.0173 (11)	0.0132 (9)	0.0134 (9)	−0.0002 (8)	−0.0001 (8)	−0.0007 (7)
N2C	0.0231 (12)	0.0202 (10)	0.0149 (10)	0.0016 (9)	−0.0042 (9)	−0.0018 (8)
C1C	0.0217 (13)	0.0173 (11)	0.0149 (11)	−0.0035 (10)	−0.0004 (10)	0.0004 (9)
C2C	0.0202 (13)	0.0146 (11)	0.0132 (11)	−0.0004 (9)	0.0005 (10)	−0.0020 (9)
C3C	0.0211 (13)	0.0131 (10)	0.0098 (10)	0.0030 (9)	−0.0010 (9)	−0.0020 (8)
C4C	0.0209 (13)	0.0135 (11)	0.0171 (12)	0.0020 (9)	−0.0022 (10)	−0.0004 (9)
C5C	0.0233 (13)	0.0170 (11)	0.0142 (11)	0.0035 (10)	0.0035 (10)	−0.0010 (9)
C6C	0.0243 (13)	0.0129 (11)	0.0131 (11)	0.0041 (9)	−0.0014 (10)	−0.0002 (8)
C7C	0.0222 (13)	0.0180 (12)	0.0160 (11)	0.0019 (10)	−0.0023 (10)	−0.0019 (9)
C8C	0.0197 (13)	0.0181 (12)	0.0163 (12)	0.0012 (9)	0.0011 (10)	−0.0018 (9)
C9C	0.0176 (12)	0.0134 (11)	0.0159 (11)	−0.0008 (9)	0.0036 (10)	0.0007 (9)
C10C	0.0324 (16)	0.0219 (13)	0.0210 (13)	0.0061 (11)	−0.0022 (12)	−0.0072 (10)
C11C	0.0368 (17)	0.0314 (14)	0.0172 (12)	0.0004 (12)	−0.0088 (12)	−0.0038 (11)
O1D	0.0256 (10)	0.0235 (9)	0.0125 (8)	0.0018 (8)	−0.0037 (7)	−0.0028 (7)
O2D	0.0313 (11)	0.0303 (10)	0.0173 (9)	−0.0020 (9)	−0.0011 (8)	−0.0096 (8)
O3D	0.0230 (10)	0.0174 (8)	0.0203 (9)	0.0024 (7)	−0.0042 (8)	−0.0027 (7)
O4D	0.0321 (11)	0.0193 (9)	0.0132 (8)	0.0038 (8)	−0.0039 (8)	−0.0038 (7)
N1D	0.0158 (10)	0.0147 (9)	0.0127 (9)	0.0005 (8)	0.0009 (8)	−0.0015 (7)
N2D	0.0232 (12)	0.0193 (10)	0.0122 (9)	0.0027 (9)	−0.0005 (8)	−0.0017 (8)
C1D	0.0195 (13)	0.0204 (12)	0.0160 (11)	−0.0013 (10)	0.0015 (10)	−0.0033 (9)
C2D	0.0196 (13)	0.0163 (11)	0.0138 (11)	−0.0004 (9)	0.0019 (10)	−0.0045 (9)
C3D	0.0186 (12)	0.0144 (11)	0.0116 (11)	0.0036 (9)	−0.0002 (9)	−0.0031 (8)
C4D	0.0271 (14)	0.0130 (11)	0.0184 (12)	0.0010 (10)	0.0007 (10)	−0.0025 (9)
C5D	0.0314 (15)	0.0163 (11)	0.0128 (11)	0.0041 (10)	0.0024 (10)	0.0024 (9)
C6D	0.0183 (13)	0.0182 (11)	0.0125 (11)	0.0051 (9)	−0.0007 (9)	−0.0029 (9)
C7D	0.0208 (13)	0.0134 (11)	0.0184 (12)	0.0027 (9)	−0.0023 (10)	−0.0040 (9)
C8D	0.0219 (13)	0.0155 (11)	0.0137 (11)	0.0040 (9)	−0.0002 (9)	−0.0002 (9)
C9D	0.0176 (12)	0.0152 (11)	0.0173 (12)	−0.0010 (9)	0.0017 (10)	−0.0028 (9)
C10D	0.0309 (16)	0.0214 (13)	0.0281 (14)	0.0056 (11)	−0.0022 (12)	−0.0105 (11)
C11D	0.0400 (17)	0.0229 (13)	0.0208 (13)	0.0026 (12)	−0.0079 (12)	−0.0062 (10)

### Geometric parameters (Å, °)

**Table:**

O1A—N2A	1.369 (3)	O1C—N2C	1.370 (3)
O1A—C1A	1.422 (3)	O1C—C1C	1.428 (3)
O2A—C1A	1.206 (3)	O2C—C1C	1.208 (3)
O3A—C9A	1.221 (3)	O3C—C9C	1.226 (3)
O4A—C6A	1.362 (3)	O4C—C6C	1.366 (3)
O4A—C11A	1.424 (3)	O4C—C11C	1.433 (4)
N1A—N2A	1.297 (3)	N1C—N2C	1.303 (3)
N1A—C2A	1.365 (3)	N1C—C2C	1.370 (3)
N1A—C3A	1.442 (3)	N1C—C3C	1.452 (3)
C1A—C2A	1.424 (3)	C1C—C2C	1.422 (3)
C2A—C9A	1.463 (4)	C2C—C9C	1.461 (3)
C3A—C8A	1.385 (3)	C3C—C8C	1.379 (4)
C3A—C4A	1.387 (3)	C3C—C4C	1.384 (3)
C8A—C7A	1.396 (3)	C4C—C5C	1.392 (3)
C8A—H8AA	0.9300	C4C—H4CA	0.9300
C7A—C6A	1.396 (3)	C5C—C6C	1.390 (4)
C7A—H7AA	0.9300	C5C—H5CA	0.9300
C6A—C5A	1.401 (4)	C6C—C7C	1.392 (4)
C5A—C4A	1.382 (3)	C7C—C8C	1.389 (3)
C5A—H5AA	0.9300	C7C—H7CA	0.9300
C4A—H4AA	0.9300	C8C—H8CA	0.9300
C9A—C10A	1.501 (3)	C9C—C10C	1.503 (3)
C10A—H10A	0.9600	C10C—H10G	0.9600
C10A—H10B	0.9600	C10C—H10H	0.9600
C10A—H10C	0.9600	C10C—H10I	0.9600
C11A—H11A	0.9600	C11C—H11G	0.9600
C11A—H11B	0.9600	C11C—H11H	0.9600
C11A—H11C	0.9600	C11C—H11I	0.9600
O1B—N2B	1.372 (3)	O1D—N2D	1.370 (3)
O1B—C1B	1.430 (3)	O1D—C1D	1.429 (3)
O2B—C1B	1.207 (3)	O2D—C1D	1.205 (3)
O3B—C9B	1.219 (3)	O3D—C9D	1.222 (3)
O4B—C6B	1.361 (3)	O4D—C6D	1.359 (3)
O4B—C11B	1.436 (4)	O4D—C11D	1.435 (3)
N1B—N2B	1.298 (3)	N1D—N2D	1.303 (3)
N1B—C2B	1.375 (3)	N1D—C2D	1.364 (3)
N1B—C3B	1.449 (3)	N1D—C3D	1.450 (3)
C1B—C2B	1.418 (4)	C1D—C2D	1.426 (3)
C2B—C9B	1.451 (4)	C2D—C9D	1.469 (4)
C3B—C8B	1.379 (4)	C3D—C8D	1.378 (3)
C3B—C4B	1.391 (3)	C3D—C4D	1.395 (3)
C4B—C5B	1.381 (3)	C4D—C5D	1.376 (3)
C4B—H4BA	0.9300	C4D—H4DA	0.9300
C5B—C6B	1.392 (4)	C5D—C6D	1.406 (3)
C5B—H5BA	0.9300	C5D—H5DA	0.9300
C6B—C7B	1.399 (4)	C6D—C7D	1.394 (3)
C7B—C8B	1.392 (3)	C7D—C8D	1.402 (3)
C7B—H7BA	0.9300	C7D—H7DA	0.9300
C8B—H8BA	0.9300	C8D—H8DA	0.9300
C9B—C10B	1.505 (3)	C9D—C10D	1.498 (3)
C10B—H10D	0.9600	C10D—H10J	0.9600
C10B—H10E	0.9600	C10D—H10M	0.9600
C10B—H10F	0.9600	C10D—H10K	0.9600
C11B—H11D	0.9600	C11D—H11M	0.9600
C11B—H11E	0.9600	C11D—H11J	0.9600
C11B—H11F	0.9600	C11D—H11K	0.9600
			
N2A—O1A—C1A	110.86 (18)	N2C—O1C—C1C	110.72 (18)
C6A—O4A—C11A	117.9 (2)	C6C—O4C—C11C	117.4 (2)
N2A—N1A—C2A	115.0 (2)	N2C—N1C—C2C	114.7 (2)
N2A—N1A—C3A	115.87 (19)	N2C—N1C—C3C	115.31 (19)
C2A—N1A—C3A	128.9 (2)	C2C—N1C—C3C	129.8 (2)
N1A—N2A—O1A	105.26 (18)	N1C—N2C—O1C	105.44 (19)
O2A—C1A—O1A	119.8 (2)	O2C—C1C—C2C	137.3 (3)
O2A—C1A—C2A	136.7 (3)	O2C—C1C—O1C	119.0 (2)
O1A—C1A—C2A	103.54 (19)	C2C—C1C—O1C	103.7 (2)
N1A—C2A—C1A	105.3 (2)	N1C—C2C—C1C	105.5 (2)
N1A—C2A—C9A	125.9 (2)	N1C—C2C—C9C	126.7 (2)
C1A—C2A—C9A	128.4 (2)	C1C—C2C—C9C	127.8 (2)
C8A—C3A—C4A	122.3 (2)	C8C—C3C—C4C	122.3 (2)
C8A—C3A—N1A	119.3 (2)	C8C—C3C—N1C	120.1 (2)
C4A—C3A—N1A	118.3 (2)	C4C—C3C—N1C	117.6 (2)
C3A—C8A—C7A	118.5 (2)	C3C—C4C—C5C	118.4 (2)
C3A—C8A—H8AA	120.7	C3C—C4C—H4CA	120.8
C7A—C8A—H8AA	120.7	C5C—C4C—H4CA	120.8
C8A—C7A—C6A	120.1 (2)	C6C—C5C—C4C	120.1 (2)
C8A—C7A—H7AA	120.0	C6C—C5C—H5CA	119.9
C6A—C7A—H7AA	120.0	C4C—C5C—H5CA	119.9
O4A—C6A—C7A	124.8 (2)	O4C—C6C—C5C	115.7 (2)
O4A—C6A—C5A	115.2 (2)	O4C—C6C—C7C	123.7 (2)
C7A—C6A—C5A	120.0 (2)	C5C—C6C—C7C	120.6 (2)
C4A—C5A—C6A	120.2 (2)	C8C—C7C—C6C	119.5 (2)
C4A—C5A—H5AA	119.9	C8C—C7C—H7CA	120.2
C6A—C5A—H5AA	119.9	C6C—C7C—H7CA	120.2
C5A—C4A—C3A	118.9 (2)	C3C—C8C—C7C	119.1 (2)
C5A—C4A—H4AA	120.6	C3C—C8C—H8CA	120.4
C3A—C4A—H4AA	120.6	C7C—C8C—H8CA	120.4
O3A—C9A—C2A	121.5 (2)	O3C—C9C—C2C	123.0 (2)
O3A—C9A—C10A	121.8 (2)	O3C—C9C—C10C	121.3 (2)
C2A—C9A—C10A	116.7 (2)	C2C—C9C—C10C	115.7 (2)
C9A—C10A—H10A	109.5	C9C—C10C—H10G	109.5
C9A—C10A—H10B	109.5	C9C—C10C—H10H	109.5
H10A—C10A—H10B	109.5	H10G—C10C—H10H	109.5
C9A—C10A—H10C	109.5	C9C—C10C—H10I	109.5
H10A—C10A—H10C	109.5	H10G—C10C—H10I	109.5
H10B—C10A—H10C	109.5	H10H—C10C—H10I	109.5
O4A—C11A—H11A	109.5	O4C—C11C—H11G	109.5
O4A—C11A—H11B	109.5	O4C—C11C—H11H	109.5
H11A—C11A—H11B	109.5	H11G—C11C—H11H	109.5
O4A—C11A—H11C	109.5	O4C—C11C—H11I	109.5
H11A—C11A—H11C	109.5	H11G—C11C—H11I	109.5
H11B—C11A—H11C	109.5	H11H—C11C—H11I	109.5
N2B—O1B—C1B	110.80 (18)	N2D—O1D—C1D	110.37 (18)
C6B—O4B—C11B	117.3 (2)	C6D—O4D—C11D	118.05 (19)
N2B—N1B—C2B	115.1 (2)	N2D—N1D—C2D	114.9 (2)
N2B—N1B—C3B	115.0 (2)	N2D—N1D—C3D	116.12 (19)
C2B—N1B—C3B	129.7 (2)	C2D—N1D—C3D	128.8 (2)
N1B—N2B—O1B	105.10 (19)	N1D—N2D—O1D	105.58 (18)
O2B—C1B—C2B	137.1 (3)	O2D—C1D—C2D	136.8 (3)
O2B—C1B—O1B	119.1 (2)	O2D—C1D—O1D	119.4 (2)
C2B—C1B—O1B	103.8 (2)	C2D—C1D—O1D	103.76 (19)
N1B—C2B—C1B	105.2 (2)	N1D—C2D—C1D	105.4 (2)
N1B—C2B—C9B	126.6 (2)	N1D—C2D—C9D	125.4 (2)
C1B—C2B—C9B	128.1 (2)	C1D—C2D—C9D	128.8 (2)
C8B—C3B—C4B	122.5 (2)	C8D—C3D—C4D	122.4 (2)
C8B—C3B—N1B	119.9 (2)	C8D—C3D—N1D	119.2 (2)
C4B—C3B—N1B	117.6 (2)	C4D—C3D—N1D	118.4 (2)
C5B—C4B—C3B	118.8 (2)	C5D—C4D—C3D	118.6 (2)
C5B—C4B—H4BA	120.6	C5D—C4D—H4DA	120.7
C3B—C4B—H4BA	120.6	C3D—C4D—H4DA	120.7
C4B—C5B—C6B	119.8 (2)	C4D—C5D—C6D	120.5 (2)
C4B—C5B—H5BA	120.1	C4D—C5D—H5DA	119.8
C6B—C5B—H5BA	120.1	C6D—C5D—H5DA	119.8
O4B—C6B—C5B	115.0 (2)	O4D—C6D—C7D	125.0 (2)
O4B—C6B—C7B	124.3 (2)	O4D—C6D—C5D	115.0 (2)
C5B—C6B—C7B	120.8 (2)	C7D—C6D—C5D	120.0 (2)
C8B—C7B—C6B	119.5 (2)	C6D—C7D—C8D	119.7 (2)
C8B—C7B—H7BA	120.2	C6D—C7D—H7DA	120.2
C6B—C7B—H7BA	120.2	C8D—C7D—H7DA	120.2
C3B—C8B—C7B	118.6 (2)	C3D—C8D—C7D	118.9 (2)
C3B—C8B—H8BA	120.7	C3D—C8D—H8DA	120.6
C7B—C8B—H8BA	120.7	C7D—C8D—H8DA	120.6
O3B—C9B—C2B	122.8 (2)	O3D—C9D—C2D	122.1 (2)
O3B—C9B—C10B	121.6 (2)	O3D—C9D—C10D	121.7 (2)
C2B—C9B—C10B	115.6 (2)	C2D—C9D—C10D	116.3 (2)
C9B—C10B—H10D	109.5	C9D—C10D—H10J	109.5
C9B—C10B—H10E	109.5	C9D—C10D—H10M	109.5
H10D—C10B—H10E	109.5	H10J—C10D—H10M	109.5
C9B—C10B—H10F	109.5	C9D—C10D—H10K	109.5
H10D—C10B—H10F	109.5	H10J—C10D—H10K	109.5
H10E—C10B—H10F	109.5	H10M—C10D—H10K	109.5
O4B—C11B—H11D	109.5	O4D—C11D—H11M	109.5
O4B—C11B—H11E	109.5	O4D—C11D—H11J	109.5
H11D—C11B—H11E	109.5	H11M—C11D—H11J	109.5
O4B—C11B—H11F	109.5	O4D—C11D—H11K	109.5
H11D—C11B—H11F	109.5	H11M—C11D—H11K	109.5
H11E—C11B—H11F	109.5	H11J—C11D—H11K	109.5
			
C2A—N1A—N2A—O1A	0.9 (3)	C2C—N1C—N2C—O1C	−0.2 (3)
C3A—N1A—N2A—O1A	176.26 (19)	C3C—N1C—N2C—O1C	175.58 (19)
C1A—O1A—N2A—N1A	−1.2 (2)	C1C—O1C—N2C—N1C	0.0 (3)
N2A—O1A—C1A—O2A	−179.0 (2)	N2C—O1C—C1C—O2C	−178.4 (2)
N2A—O1A—C1A—C2A	1.0 (3)	N2C—O1C—C1C—C2C	0.2 (3)
N2A—N1A—C2A—C1A	−0.3 (3)	N2C—N1C—C2C—C1C	0.4 (3)
C3A—N1A—C2A—C1A	−174.9 (2)	C3C—N1C—C2C—C1C	−174.7 (2)
N2A—N1A—C2A—C9A	−173.7 (2)	N2C—N1C—C2C—C9C	179.5 (2)
C3A—N1A—C2A—C9A	11.7 (4)	C3C—N1C—C2C—C9C	4.4 (4)
O2A—C1A—C2A—N1A	179.5 (3)	O2C—C1C—C2C—N1C	177.9 (3)
O1A—C1A—C2A—N1A	−0.4 (2)	O1C—C1C—C2C—N1C	−0.3 (3)
O2A—C1A—C2A—C9A	−7.3 (5)	O2C—C1C—C2C—C9C	−1.2 (5)
O1A—C1A—C2A—C9A	172.7 (2)	O1C—C1C—C2C—C9C	−179.4 (2)
N2A—N1A—C3A—C8A	56.4 (3)	N2C—N1C—C3C—C8C	122.4 (2)
C2A—N1A—C3A—C8A	−129.0 (3)	C2C—N1C—C3C—C8C	−62.5 (3)
N2A—N1A—C3A—C4A	−121.6 (3)	N2C—N1C—C3C—C4C	−55.9 (3)
C2A—N1A—C3A—C4A	53.0 (4)	C2C—N1C—C3C—C4C	119.1 (3)
C4A—C3A—C8A—C7A	−0.1 (4)	C8C—C3C—C4C—C5C	0.5 (4)
N1A—C3A—C8A—C7A	−178.0 (2)	N1C—C3C—C4C—C5C	178.8 (2)
C3A—C8A—C7A—C6A	−1.5 (4)	C3C—C4C—C5C—C6C	0.2 (3)
C11A—O4A—C6A—C7A	0.9 (4)	C11C—O4C—C6C—C5C	177.5 (2)
C11A—O4A—C6A—C5A	−179.0 (3)	C11C—O4C—C6C—C7C	−2.5 (3)
C8A—C7A—C6A—O4A	−177.8 (2)	C4C—C5C—C6C—O4C	179.7 (2)
C8A—C7A—C6A—C5A	2.0 (4)	C4C—C5C—C6C—C7C	−0.3 (4)
O4A—C6A—C5A—C4A	178.9 (2)	O4C—C6C—C7C—C8C	179.7 (2)
C7A—C6A—C5A—C4A	−1.0 (4)	C5C—C6C—C7C—C8C	−0.3 (4)
C6A—C5A—C4A—C3A	−0.6 (4)	C4C—C3C—C8C—C7C	−1.1 (4)
C8A—C3A—C4A—C5A	1.1 (4)	N1C—C3C—C8C—C7C	−179.4 (2)
N1A—C3A—C4A—C5A	179.1 (2)	C6C—C7C—C8C—C3C	1.0 (4)
N1A—C2A—C9A—O3A	7.0 (4)	N1C—C2C—C9C—O3C	7.1 (4)
C1A—C2A—C9A—O3A	−164.9 (2)	C1C—C2C—C9C—O3C	−174.0 (2)
N1A—C2A—C9A—C10A	−173.7 (2)	N1C—C2C—C9C—C10C	−173.2 (2)
C1A—C2A—C9A—C10A	14.5 (4)	C1C—C2C—C9C—C10C	5.7 (4)
C2B—N1B—N2B—O1B	0.2 (3)	C2D—N1D—N2D—O1D	−1.4 (3)
C3B—N1B—N2B—O1B	175.62 (19)	C3D—N1D—N2D—O1D	−176.18 (19)
C1B—O1B—N2B—N1B	−0.9 (3)	C1D—O1D—N2D—N1D	1.7 (3)
N2B—O1B—C1B—O2B	−177.6 (2)	N2D—O1D—C1D—O2D	178.9 (2)
N2B—O1B—C1B—C2B	1.2 (3)	N2D—O1D—C1D—C2D	−1.4 (3)
N2B—N1B—C2B—C1B	0.5 (3)	N2D—N1D—C2D—C1D	0.6 (3)
C3B—N1B—C2B—C1B	−174.0 (2)	C3D—N1D—C2D—C1D	174.5 (2)
N2B—N1B—C2B—C9B	179.3 (2)	N2D—N1D—C2D—C9D	174.0 (2)
C3B—N1B—C2B—C9B	4.7 (4)	C3D—N1D—C2D—C9D	−12.1 (4)
O2B—C1B—C2B—N1B	177.4 (3)	O2D—C1D—C2D—N1D	−179.8 (3)
O1B—C1B—C2B—N1B	−1.0 (2)	O1D—C1D—C2D—N1D	0.5 (3)
O2B—C1B—C2B—C9B	−1.3 (5)	O2D—C1D—C2D—C9D	7.1 (5)
O1B—C1B—C2B—C9B	−179.7 (2)	O1D—C1D—C2D—C9D	−172.6 (2)
N2B—N1B—C3B—C8B	119.8 (3)	N2D—N1D—C3D—C8D	−55.2 (3)
C2B—N1B—C3B—C8B	−65.6 (3)	C2D—N1D—C3D—C8D	130.9 (3)
N2B—N1B—C3B—C4B	−58.0 (3)	N2D—N1D—C3D—C4D	122.5 (3)
C2B—N1B—C3B—C4B	116.6 (3)	C2D—N1D—C3D—C4D	−51.4 (4)
C8B—C3B—C4B—C5B	0.6 (4)	C8D—C3D—C4D—C5D	−1.2 (4)
N1B—C3B—C4B—C5B	178.3 (2)	N1D—C3D—C4D—C5D	−178.8 (2)
C3B—C4B—C5B—C6B	0.2 (4)	C3D—C4D—C5D—C6D	1.0 (4)
C11B—O4B—C6B—C5B	−179.9 (2)	C11D—O4D—C6D—C7D	0.7 (4)
C11B—O4B—C6B—C7B	0.2 (4)	C11D—O4D—C6D—C5D	−178.7 (2)
C4B—C5B—C6B—O4B	179.7 (2)	C4D—C5D—C6D—O4D	179.6 (2)
C4B—C5B—C6B—C7B	−0.4 (4)	C4D—C5D—C6D—C7D	0.2 (4)
O4B—C6B—C7B—C8B	179.7 (2)	O4D—C6D—C7D—C8D	179.4 (2)
C5B—C6B—C7B—C8B	−0.1 (4)	C5D—C6D—C7D—C8D	−1.2 (4)
C4B—C3B—C8B—C7B	−1.2 (4)	C4D—C3D—C8D—C7D	0.2 (4)
N1B—C3B—C8B—C7B	−178.9 (2)	N1D—C3D—C8D—C7D	177.8 (2)
C6B—C7B—C8B—C3B	0.9 (4)	C6D—C7D—C8D—C3D	1.0 (4)
N1B—C2B—C9B—O3B	8.2 (4)	N1D—C2D—C9D—O3D	−6.4 (4)
C1B—C2B—C9B—O3B	−173.4 (2)	C1D—C2D—C9D—O3D	165.4 (3)
N1B—C2B—C9B—C10B	−172.0 (2)	N1D—C2D—C9D—C10D	174.0 (2)
C1B—C2B—C9B—C10B	6.4 (4)	C1D—C2D—C9D—C10D	−14.2 (4)

### Hydrogen-bond geometry (Å, °)

**Table:**

*D*—H···*A*	*D*—H	H···*A*	*D*···*A*	*D*—H···*A*
C5A—H5AA···O3B^i^	0.93	2.49	3.282 (3)	144
C4B—H4BA···O3A	0.93	2.48	3.217 (3)	137
C8B—H8BA···N2A^ii^	0.93	2.47	3.369 (4)	164
C4C—H4CA···O3D^iii^	0.93	2.49	3.231 (3)	137
C11B—H11D···O2D^ii^	0.96	2.39	3.220 (4)	144
C11C—H11G···O2B^iv^	0.96	2.47	3.289 (4)	143
C8C—H8CA···N2D^v^	0.93	2.47	3.386 (4)	169
C5D—H5DA···O3C^vi^	0.93	2.56	3.311 (3)	138

Symmetry codes: (i) −*x*+1, −*y*, −*z*+1; (ii) *x*−1, *y*, *z*; (iii) −*x*+2, −*y*+1, −*z*+1; (iv) −*x*+1, −*y*, −*z*; (v) −*x*+1, −*y*+1, −*z*+1; (vi) *x*, *y*, *z*+1.
